# Using modified grounded theory for conducting systematic research study on sustainable project management field

**DOI:** 10.1016/j.mex.2022.101897

**Published:** 2022-11-01

**Authors:** Moawiah A. Alnsour

**Affiliations:** Assistant Professor of Project Management and Sustainability, Department of Civil Engineering, Isra University, Amman, Jordan

**Keywords:** Sustainability, Project management, Modified grounded theory, Jordan

## Abstract

This paper introduces a method to conduct systematic research using modified grounded theory (MGT) in sustainable project management as an extended version of classical grounded theory. MGT allows for data collection from responders, followed by data analysis to generate findings into a set of categories. Recently, academic papers have revealed a lack of systematic approaches to conducting research in the sustainable project management field, an increasingly important area of study. The current research employs a method to investigate the sustainability practice in Jordan and the complexities and capabilities of sustainable project management. However, despite previous efforts within sustainable project management, researchers still seek consensus on the methodology for its consideration. Therefore, this study describes a novel step-by-step approach for conducting research to achieve this goal. Sustainable project management is challenging since it tends to be either qualitative or quantitative; hence, the identification of the appropriate research method is a hard task for a researcher. As a result, several research methods are often employed, and MGT is introduced as an effective approach. Indeed, since sustainable project management is an emerging field in Jordan, adopting a new research method should be considered over traditional approaches.

• *This paper introduces a new approach to investigating complexities and capabilities within sustainable project management.*
• *The paper presents a brief description of the microanalysis of qualitative data.*
• *The method establishes guidelines for generating theories from the ground up.*

## Graphical abstract

**Figure:**
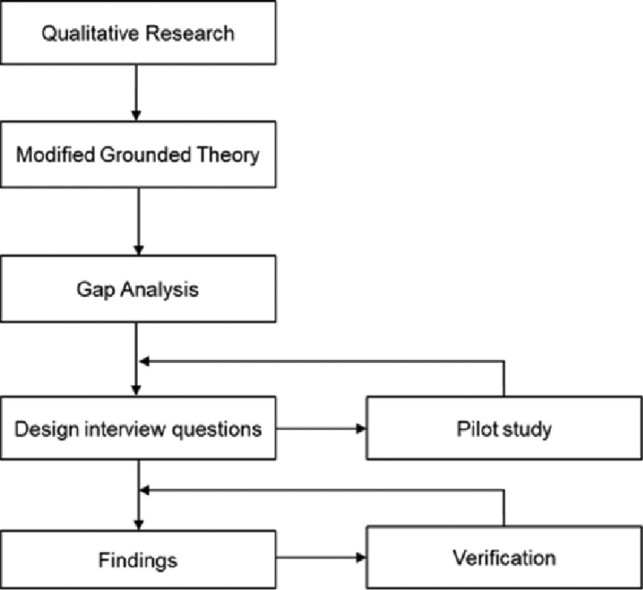

Specifications Table

**Table:** 

Subject area	Sustainability
Specific subject	*Project Management Field*
Method name	Modified grounded theory
Name and reference of original method	*Grounded Theory* Glaser, *B*. and *A.L*. Strauss, *G. & Strauss, A., L. (1967). The Discovery of Grounded Theory: Strategies for Qualitative Research*, in *Aldine Transaction A Division of Transaction Publishers New Brunswick (USA) and London (UK)*. Corbin, *J.M*. and *A*. Strauss, *Grounded theory research: Procedures, canons, and evaluative criteria.* Qualitative Sociology, 1990. 13(1): p. 3–21.
Recourse Availability	*Not applicable*

## Background

Research is a systematic process of studying materials to establish facts and devise conclusions [1]. From this definition, new knowledge in the field of study is essential [1]. In this regard, grounded theory (GT) is an effective method to make a significant contribution to areas that have little research. Therefore, the current study employs this traditional methodology, which was developed by Strauss and Corbin in 1967. While the GT method generates theory, it can also be used to investigate a subject that has yet to be researched. Correspondingly, little research has been conducted in the sustainable project management field, which is currently a hot topic. In fact, interest in adopting sustainability practices in project management is increasing worldwide [2,3]. However, in developing countries, the interest in adopting sustainability practices in project management has been of lower priority [4,5]. Although the literature regarding sustainable project management has grown, clear evidence is lacking in developing countries as this subject is relatively new [3,^4^,6].

Sustainable development is an emerging field that focuses on how we live. Seventeen Sustainable Development Goals (SDGs) have been adopted, which aim to improve life on this planet [5]. As a result, many studies have been carried out to help achieve these goals. Some research groups focus on clean water (SDG 6), while others investigate opportunities with energy, climate change, and poverty [5]. A clear methodology for attaining these goals in a balanced way has not been established. Although numerous efforts have been conducted in the sustainable project management field, they mostly focus on the technical side of sustainable development. In other words, these methods aim to adopt technologies, practices and tools to reduce the negative impacts on the environment, e.g. using clean energy. However, GT has received little research attention in this field, indicating more studies are needed. Despite this, GT shows promise in the sustainable project management field. As a developing country, Jordan struggles to achieve sustainable development. In fact, a clear and systematic method for conducting research in the field is needed. Therefore, this study conducts systematic research to contribute to the sustainable project management field. It introduces a methodology for qualitative research using the modified grounded theory (MGT) that allows practitioners and analysts to fully understand how to perform certain research.

According to the literature, a qualitative approach has many features that align with this research, including attitude measurements based on opinions, views, and close perceptions. In this case, the research is based on respondents’ opinions, beliefs, and insights used to develop a theory or phenomenon [7]. Furthermore, a qualitative approach can yield primary, rich data required for an in-depth investigation and a deep understanding of sustainable project management in Jordan since the literature on the subject is limited. Finally, Gannon and Smith [8] stated that the research problem is derived from the ‘real world’. In other words, qualitative approaches need to be conducted to obtain real-world answers to actual problems, which are not extracted using a quantitative approach [9]. As a result, GT employs qualitative data to answer real-world problems in the field under study.

## Modified grounded theory

GT is a systematic method developed by Glaser and Strauss, whose first book was published in 1967 and entitled, ‘Discovery of Grounded Theory’ [10]. Their GT is a qualitative method in which data are gathered and analysed with a systematic set of research processes to derive theories about phenomena [11,12]. In the classic GT approach, the researcher starts the investigation with pure data with no available theoretical background or framework about the phenomena [13], which seems like a difficult feat. However, Gill and Johnson [14] argued that if theories and rough definitions exist for the phenomena prior to the fieldwork investigation, an explanation of relationships between the theories can be determined, and new relationships can be established.

As mentioned, sustainable project management is an emerging field not yet employed in Jordan. Therefore, using the modified version of GT allows us to study previous efforts in the field under investigation, compare them to the existing studies in the country and then visualise where and what issues need to be investigated. This combination of GT and theoretical background is called MGT. In this method, the researcher begins the investigation of the existing theory and builds the study based on that theory [15], [16], [17]. The MGT procedures are summarised in Table 1.

**Table 1:** MGT procedures.

Action	Remarks
Data collection	MGT interviews of the data source are employed. The data analysis begins after the first interviews of data collection [18].
Data transcription	All provided data are fully recorded after each interview to prepare for analysis [12,18].
Open coding	This is the process of disaggregation of the provided data into units [12,17]. As a result, all provided data need to be broken down into incidents and labelled into categories [12].
Theoretical saturation	At this stage, no provided data are related to any category, where categories have become well understood and developed in their relationships [17].
Abstract definition	All generated categories are developed from similar conceptual terms. They can be grouped under more abstract concepts, which are the outcomes of experiences, events and actions [17]. The main aim of naming a phenomenon is to enable the researcher to define the categories and group them under common classification headings [17].
Constant comparison	This form of comparison uses comparative analysis to break down the data and dig deeper into meanings [15]. Categories are compared, contrasted, and classified into themes [16].
Theoretical sampling	During the data collection, the interviewees should be known by the subsequent interviews to determine who will collect the necessary data for further investigation [15,16].
Axial coding	This is the process of recognising the relationships of categories to their subcategories and linking categories based on properties and dimensions. This is termed ‘axial’ because coding occurs around the category's axis [17].
Core/central category and theoretical integration	The core category emerges amongst identified categories to which all other categories are related [18]. The integration of all emerged categories around a central one begins early in the analysis and ends after the writing is complete [18]. The main central phenomena are represented by the core category [18].
Selective coding	This is ‘the process by which all categories are unified around a “core” category, and categories that need further explication are filled in with descriptive detail’ [[12], p.14]. All the emerging categories can be integrated along the dimensions to form the relationships between each category and fill any gaps that require modification [18].

In MGT, the theory is continuously developed by generating a series of observations [13]. An overall theory can be built by generating theories from collected data. Data collection and theory generation are intertwined in this process [7,13]. The analysis begins with the first interview and ends after the researcher leaves the research site. This comprehensive and systematic process starts by collecting data, analysing the data subsequentially, devising questions, asking the questions, and generating theories. The fieldwork efforts of data collection and analysis proceed simultaneously.

The first step of MGT is open coding, which is defined as ‘the analytical process where the collected data are broken down. This is the first stage of analysing qualitative data’ [12]. The collected data is disaggregated into conceptual units and provided with a label [13]. During the analysis, the label is given to similar data, which is the start of open coding. In this step, the similarities and differences are compared for the events, actions and interactions. In this way, the labels are assigned to categories and subcategories [12]. Moreover, Strauss and Corbin [17] stated that different sources derive the names for categories. Open coding discovers names and categorises phenomena in terms of their properties and dimensions until the saturation of categories is reached.

Continuing the process of analysis, axial and selective coding is needed. In this stage, data are reassembled about the nature of relationships through statements amongst the various categories and subcategories [17], commonly referred to as ‘hypotheses’. Strauss and Corbin [17] stated that, during the coding processes, the data broken down with open coding begins to reassemble with axial coding. In open coding, the relationships between categories emerge, while axial coding works further on this categorisation task [19]. In particular, the researcher needs to code around a single category, and a dense texture should be built [20].

Axial coding involves several basic tasks [19]:

• Outline the categories’ properties and dimensions.
• Identify the range of actions associated with phenomena.
• Link categories to their subcategories based on their relationships.
• Clarify how categories and subcategories can relate to each other.

Once the categories have been generalised and their relationships created, we then move on to form the theories. At this stage, selective coding takes place, which typically occurs in the later phases of a study [12]. In this case, once the central category is established, other categories are related to it through relationship statements. At this point, the sampling becomes a very careful process in which the researcher chooses sites, persons and documents that will maximise opportunities for comparative analysis and fill gaps in any categories that need modification [17].

## Research steps

In this MGT strategy, existing practices were employed to explore the gaps in Jordan's practices and overcome such complexities that hinder the sustainability of project management in Jordan. First, the current study applied existing practices to visualise the thinking and understanding of sustainable project management. However, the available international theories of sustainable project management were remote and abstract; they needed to be generalised for the context of Jordan (the country under study). In addition, the practices needed to be customised in detail. Moreover, the fieldwork investigation in Jordan was built upon the data analysis gained from current sustainable project management approaches compared with Jordan's existing practices.

### Data collection techniques

Data collection involves the communication process between the provider (respondents) to the collector (researcher) [1]. The data collected from respondents are affected by some constraints, including time and data availability. Data collection can be classified as primary or secondary [1]. An in-depth investigation of research employs interviews to obtain a deep understanding of the current situation of the research area. The interviews provide extensive pictures and rich data [1] and tend to be considered part of the qualitative approach, which develops rather than tests a theory. Furthermore, face-to-face interviews, which are better in achieving the research aim, are conducted with those having extensive experience in the field of the study. In addition, face-to-face interviews can give an in-depth picture to help the researcher understand the current situation and satisfy the research objectives. Therefore, this data collection method was suitable for this research to collect primary, rich and deep data. On the other hand, archival documents could be taken as secondary data [1]. This approach enables triangulation between data gathered from interviewees and verifies the provided information by checking back through the data collection. Therefore, this technique was used in addition to semi-structured interviews.

### Sample size

The data collection technique that is well-suited in dealing with qualitative research is based on face-to-face interactions with participants, semi-structured interviews or documentation sources. MGT, which is suited for qualitative research, requires the identification of the sample of participants who are suitable for the research according to a semi-structured interview. In this case, ‘theoretical sampling’ and ‘snowball sampling’ were employed to decide which respondent was needed for each interview question. Therefore, a preliminary sample was contacted to conduct the first set of interview questions, and the face-to-face interviewees were identified. This task ensures the current interview affects the subsequent interview, steering the researcher in the right direction. According to Johanson and Brooks [21], most research scholars do not require a specific sample size for the GT in qualitative research unless theoretical saturation is achieved. This research, therefore, conducted seven interviews with well-known participants in the field under study. The sample size could be affected by the quality of provided data from the interviews. In other words, during the theoretical sampling procedures, the researcher chose the interviewees with good reputations in the current research field. Therefore, the variety of responses could triangulate the collected data and verify its consistency, filling any gaps left by any group of interviewees.

### Gap analysis

Gap analysis can be used to compare the current situation with the desired one [22]. As a result, many issues arise, requiring clarification. Moreover, these issues can be undertaken as a start for data collecting, enabling the interplay between data collection and data analysis. In addition, new theories can emerge from an in-depth investigation, and new links between the categories can be identified. The collected data are categorised and labelled, and the relationships are identified according to the generated categories. Then, the gap practices are clarified – in the Jordanian context, in this case – to design the interview questions.

Gap analysis provides theoretical implications and determines the main issues that need to be investigated further. In this study, it explored where major improvements were needed for compliance with the relevant practices of sustainable project management worldwide. Additionally, the delivery environment of projects and sustainability were studied and considered external or internal. Moreover, the capability gaps and weaknesses of the existing practices of the organisation were evaluated. As a result, three sections during the interview questions were covered with respect to the sustainable project management field:

• Sustainability practices in Jordan
• Complexity
• Capability

### Development of subsequent interview questions

#### Step 1. Pilot the derived fixed research questions

After the gap analysis, we derived several questions that were related to the implications of the analysis results. These questions could not be asked directly without testing their reliability and development. In fact, a pilot study is a testing step of a particular research instrument used to obtain feedback from the respondents [1]. The feedback gives the opportunity to improve the interview questions, fill in gaps and identify the time required for completing the interview [1]. A pilot study was conducted with a small group of experts in sustainable project management. This small-scale sample trial examined whether the interview questions were fully understood and easy to answer, and the results indicated any possible problem or failure that could happen during the study. This stage established propriety before conducting the fieldwork interviews in Jordan. It explored the weaknesses and gaps in the research design and areas for improvement to any problem question. When testing the interview questions, we aimed to do the following:

• Confirm that the protocol of conducting the interviews would be followed consistently.
• Examine where the research methods might be inappropriate, unclear or too complicated for this type of research.
• Learn how to deal with the interviewees effectively, including appropriate wording, physical observations and overall interview activity.
• Estimate how long the field interviews would take by recording the pilot interviews and assess if the time required is reasonable.
• Improve the interview questions based on the feedback.

#### Step 2. Conduct two-stage interviews (open and axial coding)

**Fig. 1:**
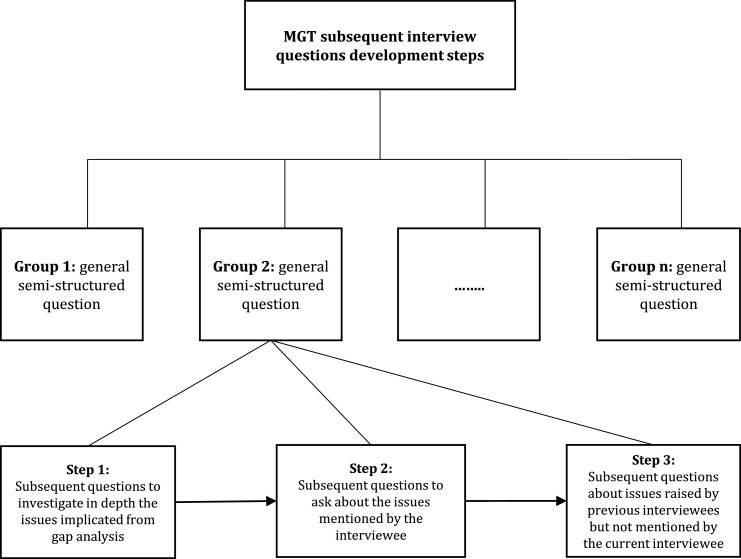
Interview groups and their development process.

In the first stage, semi-structured interviews were employed in the current research for open and axial coding for the different interview groups derived from the gap analysis. The interview questions focused on sustainability practices in Jordan, capability, and complexity. Therefore, the second-stage intensive questions were asked according to theoretical implications from existing theories through gap analysis, preliminary interviews and their implication for the next interviews. Fig. 1 shows the development process used for designing the interview questions.

The structure of the fixed general questions was derived from the sustainability analysis of the existing practices in Jordan. The gap analysis provided an overall picture to direct the fieldwork, which facilitated the design of the interview questions and testing of the existing theories in the Jordanian context. During the fieldwork in Jordan, the researcher conducted formal interviews. The interviewees were encouraged to speak freely about the topic to obtain in-depth information on the research topic and harness any new ideas not evident from the gap analysis. To reduce bias, the process could not guide the interviewees based on the research goals while allowing them to discuss openly in the field. Each group of questions included three interview steps. Step 1 involved the questions derived from the main issues explored through the gap analysis for further investigation through interviews. These issues drove the data collection using categories that emerged from the fieldwork and practical information that could later be collected from the in-depth interviews. This step allowed the interviewees to give opinions and judgements during the fieldwork, yielding valuable information for further investigation into the existing practices of sustainability.

Step 2 is an in-depth investigation of the issues mentioned by the interviewees that did not emerge during the gap analysis. This step was crucial to test the existing practices in the context of Jordan and obtain further information compatible with the gap analysis and prior interviews. Additionally, the interviewee’ experiences, judgements, and knowledge could be used to generate as many categories as possible. Step 3 verifies that the findings from the theoretical implications through the gap analysis and interviews could enable additional relevant data to be collected.

During the interview process, the collected data were analysed once the interview was complete. The information was then categorised and labelled based on the interview source, facilitating subsequent reviews of the interview data for any missing or unclear information. In addition, this process is necessary when the categories are not well defined, and an interview needs to be reviewed to clarify the category. In some cases, some categories might not be fully developed during the open and axial coding; therefore, the selective coding process further develops these categories. Each category was recorded based on the interview source to also prepare for the subsequent interview questions. Each question could involve several categories, while each category had specific properties and dimensions. These categories were generated as much as possible until a theoretical saturation for each category was achieved. At this point, no more new information would be needed since the interviewees would repeat information.

#### Step 3. Design the open and axial coding interview questions

Strauss and Corbin [17] stated that GT begins by developing a list of interview questions related to the topic to answer the overall research question. The initial list of interview questions or areas of observation may be based on concepts derived from literature or experience or from preliminary fieldwork [17]. Therefore, the early concepts are considered a start for data collection. In fact, these early concepts can direct the researcher, but they are discarded once the data are collected. In this study, basic information was first collected regarding the interviewees’ profiles. These questions could be outlined in terms of interviewee experience, position, and type of work. The three groups of questions considered the core of the research are detailed in the appendix and summarised as follows:

• General questions about *sustainability in Jordan* to confirm that sustainability is not part of project management in Jordan and develop a set of sustainability requirements.
• Questions regarding *complexity*, including project delivery environment in terms of the availability of natural resources and energy, strategies/policies/objectives, stakeholder relationships and organisation structure.
• Questions regarding the *capability* of the client, sponsor, supplier, and market to align behaviours and identify gaps.

These three groups of questions are the core element of the interviews to investigate a way to conduct research in sustainable project management in Jordan. In addition, sub-questions were built based on these. These lists of questions were designed from the gap analysis, which was conducted to explore the gaps between the existing practices of conducting sustainability in Jordan and the international practices of sustainable project management. Reflections from each question group needed to be clarified, along with their implications on the development of the interview questions. The findings from each group were linked to the aim of this research. At each stage, the findings were reflected in the overall process of developing the subsequent questions. However, the findings showed a need for an entire effective process with necessary improvements.

The first group of questions aimed to confirm that sustainability practices are not part of project management in Jordan. Moreover, the goal was to understand why. Therefore, in-depth follow-up questions were asked based on the responses in the initial interviews. The list of questions was built, and sub-questions were then derived from those responses. Example questions were as follows:

• Why is sustainability not embedded into project management practices in Jordan?
• What are the advantages and disadvantages of emending sustainability into project management?
• Why is the cost of sustainability considered an issue for project management?
• How do you determine the need for project management?
• At which stage do you think the integration of sustainability in project management is important?
• Why does sustainability need to be integrated into this stage?
• Who ensures the strategic alignment between sustainability objectives and the organisation's objectives?
• How and when do you achieve strategic alignment between them?
• Can a process achieve this alignment effectively?
• What sustainability requirements are needed in terms of environment, society and economy, and at which stage should they be integrated?

The last question aimed to have the sustainability requirements set out in the strategic objectives of the organisation without any separation. Environmental, social and economic requirements were identified to be considered in project management. These provide general information for ‘who’, ‘why’ and ‘when’ questions regarding sustainable project management in Jordan. As a result, this can yield clear ‘why’ and ‘what’ questions for the rest of the interviewees. The gap analysis found that sustainability is not part of project management in Jordan and that projects are traditionally managed. However, the analysis indicated that sustainability is part of project management in many other countries from the onset of project development, which needs to be considered in the Jordanian context. Therefore, a group of questions was developed to embed these practices of sustainability into project management in Jordan, and project managers in the country were interviewed. To achieve triangulation amongst these interviewees and the collected data from other groups, they were asked subsequent questions to develop ways to improve the overall process of project management with respect to sustainability.

The second group of questions was about the assessment of complexities (barriers) in project management in Jordan. This group of questions was the first step in the assessment of the organisation and project delivery environment (internal and external). Many factors act as internal or external barriers to conducting sustainable project management in Jordan – stakeholder engagement, interfaces, technology requirements, and financial impact. Assessing the complexity was essential to clarify the risks and potential revenues of various opportunities. Additionally, the current conditions of the environment delivery and organisation structure could render the adoption of sustainability into project management in Jordan challenging. These are the main assessment elements such that the project is delivered under the sustainability requirements. The assessment can be divided into two sub-groups of questions: (i) the internal and external factors affecting sustainability and (ii) the legal and institutional frameworks towards the strategic alignment of the organisation's objectives and sustainability. The gap analysis indicated that the strategic objectives of sustainability were poorly integrated with the organisation's objectives, overlooking environmental, social, and economic aspects. The existing practices showed that the internal and external barriers were the drivers for complexities, such as water sources, energy environment, and funding. Moreover, administrative measures and the bureaucratic system are the main drivers of the organisation. Therefore, another set of interview questions was derived for the investigation:

• When do these barriers need to be assessed during the strategic planning process?
• How do you overcome these barriers?
• Who is responsible for this role?
• How do you ensure the current organisation conditions are appropriate for adopting sustainability? Who is in charge of this task?
• What measures are needed for the strategic alignment of the organisation's objectives and sustainability objectives?
• Why are these measures needed? When are they needed? How are these measures adopted, and by whom?
• To what extent is the current top-down approach adequate in meeting the sustainability requirements?

**Fig. 2:**
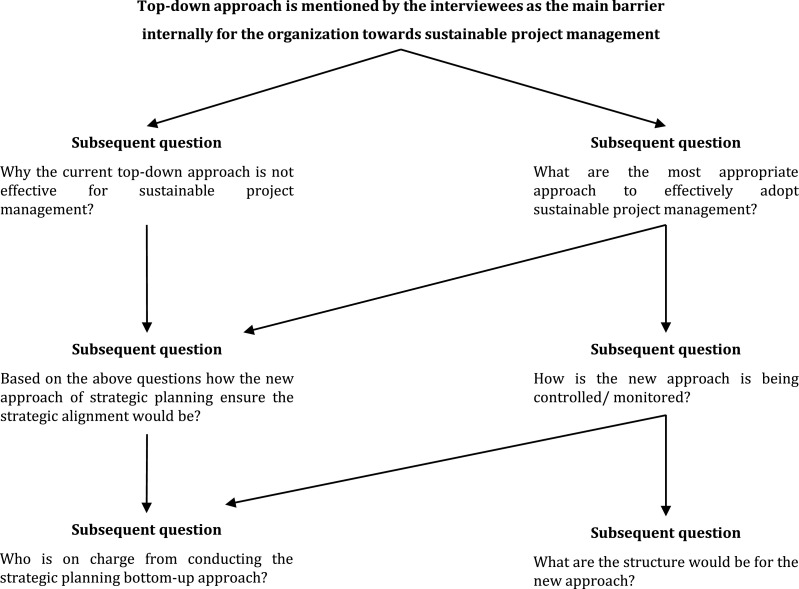
Example for subsequent questions relating to an issue.

This second subset of questions yielded an in-depth investigation of the legal and institutional frameworks. Moreover, it determined how the legal and institutional frameworks could achieve the strategic alignment of the organisation's objectives and on-ground reality. Additional questions were asked, such as ‘Do you think there is a need to update the strategic plan as the current frameworks are not stable? If yes, when does the update occur, and by whom?’ For example, Fig. 2 describes this approach to subsequent questions for the second sub-group. The top-down approach is mentioned by the interviewees as the main internal barrier for the organisation toward sustainability. The questions ‘why’ and ‘how’ revealed the relationships between these factors and ways to overcome these barriers. The respondents explained how this approach influences the key decisions that arise in each strategic planning development stage.

Another issue that can be outlined is the lack of stakeholder engagement. Therefore, the stakeholders need to be engaged from the start of strategic planning. A set of subsequent questions was designed, including the following:

• Why do stakeholders not engage in adopting sustainability into project management?
• How should the stakeholder engagement look?
• When is the integration needed? How can relationships be established between stakeholders?
• What is the purpose of engaging stakeholders? Who are they
• What tools are needed to engage stakeholders effectively?
• Do stakeholders affect the key decisions? How? What decisions can they make?

The interviewees indicated another issue regarding complexities that the sustainability objectives that come from the strategic planning process through a bottom-up approach achieve only partial sustainability. Moreover, these objectives should be comprehensive and cover all the requirements of sustainability. Therefore, a set of subsequent questions that were designed include the following:

• What are the advantages and disadvantages of developing an overall comprehensive strategic plan?
• Who will oversee the delivery of these plans? When?
• Are there any policies from the government? If so, at which level are they?
• How effective are they? Do these policies affect the comprehensive plan?
• Do you believe the integration of these policies needs to be checked?
• Who checks if these policies are effective?
• Does a monitoring system for adopting these policies exist?
• How effective are they towards sustainability?

Another barrier occurs during the strategic decision-making process. Decisions are made by the person who assumes the overall responsibility at the top management level. Therefore, the subsequent questions included the following:

• Who owns these decisions?
• Which level of management should make these decisions to achieve sustainability?
• What are the decisions, and when are they being made? What problems arise with these decisions?
• To what extent are these decisions effective toward sustainability?
• What measures are needed to improve these decisions toward sustainability?

In addition, the findings outlined that the lack of coordination between the organisations might result in misalignments between them. This issue, as mentioned by an interviewee, outlined the following set of subsequent questions, for example:

• What is the main reason for the misalignment?
• How do you then achieve coordination amongst the organisations?

The main question, then, is if the current institutional framework is effective for establishing sustainability in project management.

• How can the key stakeholders take the role of controlling/monitoring the process of sustainable project management in Jordan?

In the gap analysis, an issue arose that the coordination between each party might be achieved by strengthening the institutional framework. Therefore, a subsequent question was, ‘What measures are needed to make the institutional framework effective?’ The answer was to create a higher-level committee. Additional subsequent questions were designed based on the implications

• Who are the members of this committee?
• Who is the head of this committee?
• What are the roles and relationships of the stakeholders in this committee?
• When is this committee needed?
• What levels of decisions will this committee make?
• How can these committees achieve strategic alignment between the organisation's objectives and on-the-ground reality?
• How will these committees be monitored?
• How can the committee verify the sustainability requirements are achieved?

**Fig. 3:**
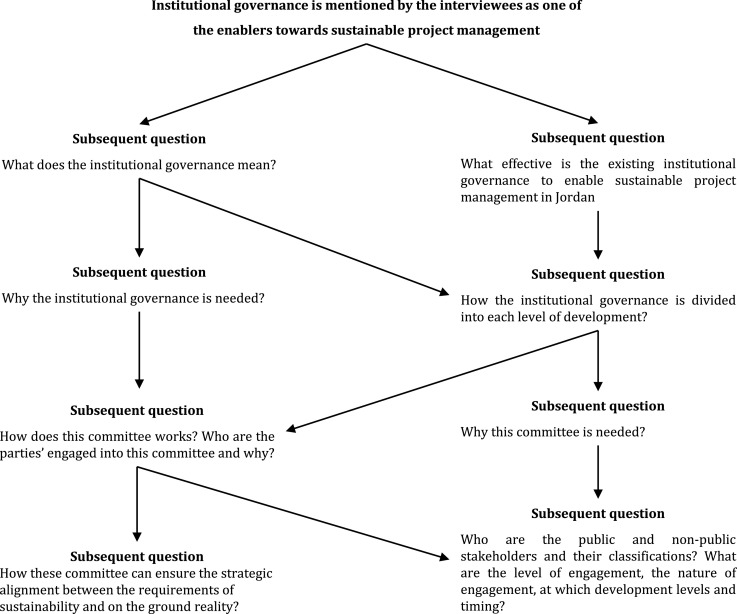
Example of subsequent questions.

In the third group of questions, an in-depth investigation was conducted to align behaviours and identify capability gaps and weaknesses of the client, sponsor, supplier, and market. In this regard, the main aim was to identify a set of programmes and best practices to enable the project's parties to establish sustainability in project management in Jordan. Moreover, this allows public organisations to confirm that all parties understand the definition of sustainability. However, the interviewees’ responses showed some weaknesses and capability gaps of the mentioned parties. As a result, such programmes should be devised for them to build up their capacity, improve their skills and understanding, and transition to a long-term perspective. From the gap analysis building team, capability is an important aspect to develop in those who are engaged in sustainability in project management. Fig. 3 shows the subsequent questions to address this issue.

Another set of questions was designed to investigate the capability gaps and weaknesses of the mentioned parties to ensure the adoption of sustainability project management in Jordan:

• Does the ministry, under the current conditions, have the capability to develop strategic objectives regarding sustainability? If yes, how? If not, where are the capability gaps?
• What skills are needed?
• How can they affect the development of the strategic objectives such that sustainability is part of project management?

Another subsequent group could be outlined in terms of the need for a specialist in sustainability:

• Are there any areas of misalignment between stakeholders, sponsors, clients, and the market?
• Do you think that the key advisor/specialist of sustainability is important to be appointed to strengthen the capability of the project's parties? If not, why?
• If yes, how the advisor/specialist plays the key role in ensuring the strategic alignment between the ministry objectives regarding sustainability and on-ground reality are realised?

These groups of questions are considered the core questions which enable us to answer the overall research question to solve the research problem. These groups of questions interplay between the data collection and data analysis for both open coding and axial coding.

#### Step 4. Design the interview questions for selective coding

Once the open and axial coding was undertaken with interviews to generate categories and investigate incidents and their relationships, selective interviews from public and private sectors and academia in strategic and project management were conducted to complete any missing or undeveloped categories and strengthen the relationships between the categories. Five groups of questions were designed for selective coding. Each includes a set of subsequent questions to investigate selected issues that had not been developed or saturated yet, determining which issues still need to be discussed. The interviewees mentioned a need to update the strategic plan and ensure that feedback from the stakeholders is acted upon. Therefore, the first set of questions outlined subsequent questions relating to this issue:

• Where in the strategic decision-planning process are the updated steps needed?
• Do you think such gateways are needed, and where?
• Do you think these gateways can affect the overall process of strategic decision-planning? What are the links between these stages?
• When can the feedback be provided? Who can give the feedback?
• When do you think the updated strategic plan is needed?

Other issues were outlined regarding committee development. The interviewees mentioned a need for a committee to manage the overall process of sustainable project management. Therefore, the relevant subsequent questions include the following:

• Do you think the higher-level committee is fully developed?
• Do you think all these parties that engaged in the higher-level committee are fully developed?
• Is any additional party needed? What are the relationships between them?
• Do you think the higher-level committee is managing the overall process of strategic planning?
• Are there any requirements?

The capability gaps mentioned by the interviewees were the major factors affecting the overall process of sustainable project management. However, we still needed to know how the committee could be assessed in terms of its understanding of the sustainability requirements and how stakeholder engagement could benefit from the team-building capacity. In addition, this group of questions could inform the relationships between the building team capacity and stakeholder engagement. The subsequent questions were designed into the open and axial coding, where further necessary information could be obtained through the selective coding process. Therefore, these questions include the following:

• At which stage do you think the building team capacity is needed?
• At which stage do you think that stakeholder identification and engagement are important?
• What tools and methods are needed to engage them?
• Do you think team-building capacity is needed when the stakeholders are engaged, and when?
• How do you ensure these are fully engaged? Do their decisions affect the overall process of strategic planning?

The following group of questions outlined any feedback and missing data that needed to be obtained. The strategic decision process of sustainable project management seemed underdeveloped and saturated during the open and axial coding, in which some issues required more details. Therefore, questions like the following were posed:

• Do you think the strategic planning stages are appropriate?
• Do you think any improvements to developed stages are needed?
• What types of evaluations are needed?
• At which stage is the most appropriate feedback needed?
• Do you think these processes serve the requirements of sustainability? Why?

Moreover, the interviewees mentioned that conducting the strength, weaknesses, opportunities, and threats (SWOT) analysis was needed at the onset of strategic decision-making. Therefore, a set of questions was designed for an in-depth investigation into such areas relating to sustainability:

• What are the main areas that need to be studied in SWOT analysis? Who performs the analysis? When?
• Do you think these areas can improve the overall sustainability practices?

All these groups from selective coding were carried out at the final stage of data collection. This process defined where more data and clarifications were needed until reaching theoretical saturation. To this end, three persons were interviewed from the public and private sectors.

## Data analysis

The starting point of the GT process is coding. Two stages were carried out to explore sustainability in project management in Jordan. The first stage of the MGT investigation was simultaneous open and axial coding. The data analysis process involved a study of the collected raw data from the interviews, and then a set of incidents were grouped into appropriate categories. The example below provides insight into how the analysis was carried out line-by-line using the microanalysis method. Two questions are listed from an interview that was conducted in Jordan, and a section of the answer is provided. The relationships between incidents and categories are identified.

***Question 1. What are the current organisational structure conditions that make the adoption of sustainability in project management complex in Jordan?***

*The current organisation's system needs to be updated regularly as the system seems inadequate for adopting sustainability. The main barrier is the personal interventions that would make the system complex. This means the top managers and key decision-makers do not want to change from traditional behaviours to new ones, and usually, the use of their powers adds complexity. One solution to overcome such complexities is to create a one-stop shop that could solve the issue and save time. The following barriers exist:*

- *Lack of coordination between each party, which causes delays in decision-making.*
- *Bureaucratic system and administrative measures.*
- *People and their culture (mentality).*
- *Lack of previous planning and poor strategic planning.*
- *Conflicts and interfaces between the interests of the organisation.*

***Question 2. How do you ensure the alignment of sustainability with the organisation's primary services or delivery objectives?***

*Moreover, the respondent explained an important aspect relating to the current institutional framework that could make the practice of sustainable project management complex. He stated that the misalignment and the missing roles amongst the institutions complicate the adoption of sustainability. He suggested developing top-down and bottom-up approaches to follow the strategy of sustainable development efficiently for sustainable project management in Jordan. The stakeholders will then take the role of evaluating the process of sustainable project management in Jordan and ensuring strategic alignment with the organisation's primary services objectives.*

**Fig. 4:**
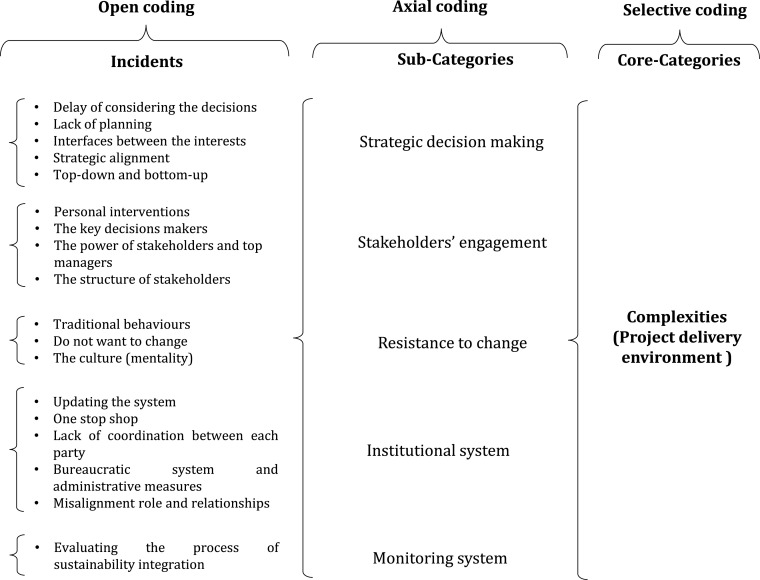
Coding example.

This response was driven by one interview, and a microanalysis was carried out during the open and axial coding process. The related data from these interviews were underlined and classified as one of the following: current organisation system, updating the system, personal interventions, key decisions-makers, do not want to change, traditional behaviours, powers of stakeholders and top managers, one-stop shop, lack of coordination between each party, delay for considering the decisions, bureaucratic system and administrative measures, culture (mentality), lack of planning and interfaces between the interests. Then, the next questions could classify some incidents as follows: misalignment role and the relationships between the institutions, top-down and bottom-up, structure of stakeholders, evaluating the process of sustainability integration, and strategic alignment of organisations objectives and sustainability. These data were coded to develop a set of categories and the relationships between them. These large datasets are labelled and grouped into appropriate categories, including the following: strategic decision-making, stakeholder engagement, resistance to change, strategic planning, institutional system and monitoring process of strategic planning. Fig. 4 shows the development process of grouping the incidents under a set of categories.

### Open coding

During the fieldwork study, the collected data from each interview was immediately analysed line-by-line using microanalysis. This process broke down the collected data into discrete parts, including concepts, incidents, and ideas. Once the researcher found that some issues needed to be confirmed by follow-up interviews, a set of subsequent questions were developed for the confirmation interview to build up the theory. Once similar incidents began to accumulate, their grouping was carried out within a specific category. They were grouped into a wide range of categories with further microanalysis, which was carried out after the interview was recorded.

### Theoretical saturation

The main goal of open coding is to generate categories and keep them open for any further changes. The researcher continued to investigate further issues until the categories were saturated. Each category grew until reaching a theoretical saturation. However, no specific sample size is defined when theoretical saturation occurs [12,17]. Therefore, such saturation was achieved once no new information was gained during the data collection process and a level of in-depth detail was obtained for categories that were realised by the researcher. Moreover, when the gathered data was repeated, the researcher stopped investigating for new information on the specific category. Theoretical sampling implies that the data are obtained from the following interviews based on the needed information. Therefore, the type of provided data steered the researcher to saturate the categories for further investigation. As a result, once several interviewees had agreed with the incidents under a set of categories, the incidents were accepted by the researcher. In the case where incidents under groups of categories were not agreed upon by the interviewees, they were rejected. The accepted incidents under specific categories were given the codes ‘P1, P2, … P7’. This process was helpful when reviewing the interviews for any missing or unclear data and checking if provided data were totally agreed upon or required further validity.

### Axial coding

Strauss and Corbin [17] stated that three sources derive the names of categories: the collected data, the terms given by the participants and the terms used by existing theories or literature. However, they claimed that the existing theories and literature could cause confusion [17] because existing terms can be associated with a reader's prior understanding of such theoretical concepts rather than the new theories’ terms [16]. Once the categories were saturated during the theoretical sampling, clear category names were assigned that would be understood by the interviewees. The categories resulting from conceptually similar terms in nature and meaning were grouped under more abstract concepts. Therefore, data broken down during open coding was reassembled in axial coding. During open coding, the relationships between categories began to emerge, while in axial coding, this task required work. This process of coding allowed the categories and their relationships to be identified. In other words, the relationships between these subcategories were tested as related to the main category. During the axial coding, the coding was performed around a single category, and a dense texture was built, and the relationships around the category's axis that shared the same properties or specific issues under the main category were given specific names:

• Strategic decision-making.
• Stakeholders’ engagement.
• Resistance to change.
• Institutional system.
• Monitoring system.

Therefore, the developed categories and their relationships were clarified based on their similarities and differences. However, the undeveloped categories in their properties, dimensions and relationships were investigated further. As a result, in-depth, subsequent interview questions were posed based on the issues obtained during the axial coding that needed further investigation.

### Selective coding

In selective coding, the poor and undeveloped categories were clarified in the second stage of data collection. The first stage of interviews was conducted with targeted samples from public and private sectors at different organisational levels. The categories at this stage were well-developed, and the relationships amongst them were well-strengthened. The poor or undeveloped categories were clarified, and the overall relationships were filled to achieve consistency and strengthen them around the central category. Once the central category was defined, the theory organised around this central category was defined. The core category was named such that all interviewees could understand it in the context of other research studies *– Complexities of delivery environment*. Furthermore, during the fieldwork, some of the sites and persons were revisited to investigate further.

Once the integrated theories were agreed upon, the process towards grounding the theory to develop an overall approach to overcome such complexities that affect the adaptation of sustainability in project management in Jordan. This was important for reaching the final level of detail, in which the integrated approach needed to be fully developed in terms of their relationships and gaps for all missing elements were filled.

## Data verification

The verification of the research indicated whether the research study's findings were reliable [1,23]. The outputs were appropriate if they matched the approximate expectations, indicating a good model [1]. The collected data were reviewed carefully to evaluate their validity and reliability, and the research interplayed between the collected data and data analysis. Therefore, during the data analysis of the interviewees’ responses, the researcher sometimes contacted interviewees for more clarification to validate the provided data. This process is critical such that the data collected is valid for the following interview. Therefore, once the analysis was carried out, the researcher prepared a set of interview questions for the subsequent interview until theoretical saturation was achieved.

## Research ethics

Research ethics is considered a crucial part of the research process. Researchers and participants need to be protected during the research. During the fieldwork, the researcher gave sufficient time, i.e., two weeks, for the interviewees to decide whether to take part in the research. Once they agreed to be part of the research, they were considered for the list of interviewees. If a potential participant provided no response, they were dropped from the list because of the time limit of this research. The decision to take part was solely that of the participant. Once agreeing to participate in this research, they gave permission to use the information they provided for research purposes only, and interview arrangements were made (time, place and date). The researcher conducted each interview. Additionally, the participant could choose if the interview was recorded. Although they were told that their responses would be kept in strict confidence, they could remain anonymous and the collected data would be anonymised and codified, they all preferred not to be recorded.

## Conclusions

This article describes steps for conducting research using MGT to investigate the existing practices, complexities and capabilities of sustainable project management in Jordan. It also tackles the data collection methods usually used for construction research. In detail, the MGT process explains how the research is conducted. The fieldwork study in Jordan provided the data through semi-structured interviews and document collection. One disadvantage of GT is that it heavily relies on the researcher's interplay between data collection and analysis. Therefore, a gap analysis was conducted to explore the existing practices of project management and sustainability in Jordan. Moreover, the initial stage of the data collection process was undertaken through a pilot study to examine the extent to which the designed set of interview questions provided the expected outcomes. Finally, the current research study proposes a methodology for conducting systematic research in the sustainable project management field that has yet to be applied in the field under study. The use of this method is recommended for similar fields since it gives the research a more solid foundation for better and more reliable results.

## Declaration of Competing Interest

The authors declare that they have no known competing financial interests or personal relationships that could have appeared to influence the work reported in this paper.

## Data Availability

Data will be made available on request.

## References

[1] Fellows R.F., Liu A.M. (2021).

[2] Armenia S., Rosa M.D., Nonino F., Pompei A. (2019). Sustainable project management: a conceptualization-oriented review and a framework proposal for future studies. Sustainability.

[3] Silvius A.G., De Graaf M. (2019). Exploring the project manager's intention to address sustainability in the project board. J. Clean. Prod..

[4] Banihashemi S., Hosseini M.R., Golizadeh H., Sankaran S. (2017). Critical success factors (CSFs) for integration of sustainability into construction project management practices in developing countries. Int. J. Proj. Manag..

[5] B. Stiftung The Sustainable Development Report. Transformation to achieve sustainable development goals includes the SDG index and dashboards (2019).

[6] Aarseth W., Ahola T., Aaltonen K., Økland A., Andersen B. (2017). Project sustainability strategies: a systematic literature review. Int. J. Proj. Manag..

[7] Bryman A., Bell E. (2007).

[8] Easterby-Smith M.T., Thorpe R., Lowe A. (2002).

[9] Yin R.K. (2003).

[10] Glaser B., Strauss A.L., Strauss A. (1967).

[11] Charmaz K. (2006). Handbook of Emergent Methods.

[12] Corbin J.M., Strauss A. (1990). Grounded theory research: procedures, canons, and evaluative criteria. Qual. Sociol..

[13] Saunders M., Lewis P., Thornhill A., Saunders M., Thornhill A., Lewis P. (2003). Research Methods for Business Students.

[14] Gill J., Johnson P. (2002).

[15] Bartlett D., Payne S. (1997).

[16] Saunders M., Lewis P., Thornhill A. (2003). Research Methods for Business Students.

[17] Strauss A., Corbin J. (1998).

[18] Corbin J., Strauss A. (2008).

[19] Strauss A.L. (1987).

[20] Bulawa P. (2014). Adapting grounded theory in qualitative research: reflections from personal experience. Int. Res. Educ..

[21] Johanson G.A., Brooks G.P. (2010). Initial scale development: sample size for pilot studies. Educ. Psychol. Meas..

[22] Neal M.A. (2019).

[23] Remenyi D., William B., Money A., Swartz E. (1998).

